# Toward Material‐Specific Peptides From in Silico Guided Peptide Engineering to Target PET in the Presence of PS

**DOI:** 10.1002/advs.77104

**Published:** 2026-08-18

**Authors:** Julian Luka, Marian Bienstein, Jonas Berger, Ulrich Schwaneberg

**Affiliations:** ^1^ Lehrstuhl für Biotechnologie RWTH Aachen University Aachen Germany; ^2^ DWI‐Leibniz Institut für Interaktive Materialien Aachen Germany

**Keywords:** material binding peptides, molecular dynamics simulations, polymer interfaces, polymer recycling, protein engineering

## Abstract

Synthetic polymers are indispensable but pose major recycling challenges, particularly in mixed plastic waste streams. Enzymatic recycling offers a promising route to specific polymer depolymerization into oligomers and monomers. This requires controlling molecular recognition at polymer interfaces. Material‐binding peptides (MBPs) can enhance catalytic degradation by directing catalysts to specific polymer surfaces, yet strategies for engineering polymer specificity remain limited. Here, we introduce a computational–experimental workflow for designing polymer‐selective MBPs. Molecular dynamics simulations identified aromatic tyrosine residues as key contributors to polystyrene (PS) binding in the MBP MacHis, while polyethylene terephthalate (PET) interactions were unaffected. Guided by these insights, targeted tyrosine‐to‐glutamate substitutions were introduced, yielding variants with an 85% reduction in PS binding while maintaining PET affinity. Steered molecular dynamics simulations confirmed the reduced PS interaction, predicting a comparable 69% decrease in binding free energy. This design principle generalized to the structurally distinct peptides LCI and Tachystatin A2, where analogous substitutions reduced PS binding by 67% or 74%, respectively. Practical application was shown by fusing peptide variants to the LCC_ICCG_ enzyme and characterizing PET degradation in mixed plastics. This workflow provides a strategy for engineering polymer‐selective binding peptides and enables programmable molecular recognition at synthetic polymer interfaces.

## Graphical abstract

This work presents a computational–experimental strategy to engineer material‐binding peptides toward polymer specificity. Molecular dynamics simulations identified residues driving polystyrene binding, enabling targeted substitutions that greatly reduced polystyrene affinity while preserving PET binding. The approach generalized across multiple peptides and was validated in enzyme fusions, providing a route to molecular recognition for enzymatic recycling of mixed plastic waste.

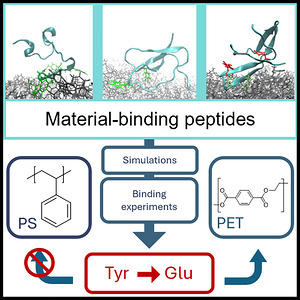

## 1 Introduction

Synthetic polymers are bulk products produced at an annual scale of 400 Mt [1]. Due to their excellent properties, including light weight, rigidity, and the ability to be formed into any shape cost‐effectively, they have become the materials of choice for many applications. Synthetic polymer products range from disposable everyday items such as food containers, cups, and cutlery to textiles and parts/devices used in transportation, building construction, and consumer goods manufacturing [2, 3]. Among synthetic polymers, mixed‐plastics containing polystyrene (PS) and polyethylene terephthalate (PET) are widely used in food packaging, including rigid clamshells, thermoformed trays, and transparent films; production of multilayer plastic packaging has been reported on a 100 Mt scale in 2018 [4]. PET and PS polymers are also commonly found in the same recycling fraction as NIR spectrometers can misclassify PS as PET [5]. Polymer recycling with respect to defined monomers and oligomers faces many challenges in sorting [6], managing food waste or other contaminants [7], and achieving economies of scale [8] so that only 9% of the produced polymers are recycled. Enzymatic polymer recycling on an industrial scale is limited by the challenges that enzymes generally do not perform well with crystalline synthetic polymers, and that melting temperatures of >240°C for PET, are beyond the thermal resistance of most PETases. As a result, industrial enzymatic PET degradation has so far been limited to pretreated mono‐material products composed of PET [9, 10, 11, 12] (commercialized CARBIOS process in 50 kt scale) [13].

Resource‐efficient recycling processes with a low carbon footprint can be envisioned through stepwise mixed‐polymer hydrolysis, yielding defined monomers and oligomers for each targeted polymer. A prerequisite for such a stepwise process is the material‐specific targeting of each polymer with a material‐specific binding peptide conjugated or fused to a chemical or enzymatic depolymerization catalyst. A stepwise depolymerization of a PET/PS mixture, for example, would start with targeted enzymatic hydrolysis of the PET polymer to recover high‐value monomers from the mixed plastic fraction before applying pyrolysis to the remaining material to depolymerize PS. Targeting of polymers with fused material‐binding peptides has been successfully reported for a cutinase [14] and inorganic Cobalt catalysts [15]. In detail, immobilization of a cutinase using the material binding peptide Tachystatin A2 enhanced the depolymerization of polyester‐polyurethan by 6.6‐fold [14] and fusing a cobalt catalyst to the material‐binding peptide LCI increased PS oxidation by 15‐fold [15] when compared to the freely supplemented cutinase or Co‐catalyst.

The interfacial design of molecular interactions between synthetic polymers and the material‐binding peptide is a key challenge for enabling sustainable processes for the recovery of defined oligomers and monomers. Computational tools can provide insights into atomic‐level interactions with all‐atom models of crystalline and amorphous PET [16, 17, 18, 19, 20] and PS [21, 22, 23, 24] already reported. One option for generating these models is the Charmm‐GUI polymer builder, which provides a generalized process for generating a variety of relaxed polymer systems from over 150 monomers, that has already been use for PS and PET models [25]. Bulk structures of synthetic polymers such as PS and PET are composed of long covalently bound macromolecular chains that become physically entangled. Entanglements are not chemical crosslinks, but topological constraints that restrict chain motion. In the amorphous phase, polymer chains typically adopt disordered, randomly coiled conformations with no long‐range periodic packing. In semi‐crystalline polymers, portions of the chains locally align and pack into ordered lamellae. Notably, entanglements are largely retained in the amorphous regions and often connect crystalline domains through tie molecules [26]. Interfacial design to develop material‐specific binding peptides for synthetic polymers such as PS and PET must account for these topological differences as well as the fundamental polymer properties.

Polystyrene (PS) is a vinyl polymer composed of repeating styrene monomers, forming a polydisperse backbone with aromatic phenyl groups as side groups. In most commercial applications, polystyrene is atactic, meaning its substituents (phenyl groups) are randomly arranged along the PS backbone. Atactic randomness disrupts chain packing and prevents regular crystallinity, resulting in an amorphous material [27]. In the amorphous state, PS molecules form a disordered structure, in which the chains interpenetrate but do not align into any long‐range, periodic arrangement. The glass transition temperature for PS generally lies around 100°C, which is above the temperature at which the polymer transitions from a rigid, glassy state to a rubbery, more malleable form [28].

Polyethylene terephthalate (PET) is a thermoplastic polyester, synthesized by the esterification of terephthalic acid and ethylene glycol. When cooled rapidly or cast into thin films, PET can exist in a purely amorphous form, exhibiting high transparency and good thermoformability. PET can also form semi‐crystalline structures if given sufficient time or thermal treatment for crystallites to grow. In its amorphous state, PET is transparent, and no long‐range order is present; polymer chains are randomly arranged with a lower modulus compared to semi‐crystalline PET [29].

Both PS and PET primarily rely on van der Waals forces and occasional π‐π interactions or dipole interactions to keep individual chains in close proximity to one another [30, 31]. In PS, the phenyl rings engage in aromatic stacking (π–π) with neighboring chains, which contributes to the polymer's relatively high glass transition temperature and stiffness [31, 32]. Since there is no possibility of hydrogen bonding in a pure hydrocarbon backbone with phenyl side groups, polystyrene's chain connectivity is largely governed by dispersion forces. In PET, carbonyl and ether groups in the backbone introduce dipole–dipole attractions, which strengthen intermolecular cohesion [33]. Under certain conditions, there can be limited hydrogen bonding with terminal hydroxyl or carboxyl end groups; however, the ester linkages might be more important due to their robust dipolar interactions [34]. Dipoles contribute to PET's relatively higher barrier properties compared to PS or other non‐polar polymers such as PP or PE. In PET's amorphous state, disordered entanglements benefit from dipolar and dispersion forces, contributing to strength and moderate thermal stability.

PET‐PS mixtures were selected, in addition to their broad applications in everyday products, for specific differences in their molecular interactions (van der Waals/π–π (PS) vs. additional dipole interactions (PET)) and in their backbone composition, which influences possible interactions and recycling options. MD simulations can elucidate molecular interactions and determine whether a specific amino acid composition can distinguish between PS and PET, which would be crucial for the interfacial design of material‐specific binding peptides.

Material binding peptides (MBPs) present a broadly applicable technology platform for the functionalization and degradation of synthetic polymers as described in a recent review by our group [35]. Binding of MBPs to polymers has been reported for PET (LCI [36], Tachystatin A2 [36], Dermaseptin S1 [37]), PS (Cecropin A^36^, LCI [36], Tachystatin A2 [36]), and several other polymers such as polylactic acid (PLA) (LCI [38], Cg‐Def [38], spinigerin [38]), polypropylene (PP) (Cecropin A^36^, LCI [36], Tachystatin A2 [36]), polyurethane (PU) (Tachystatin A2 [14]). Enhanced degradation by a fusion protein (MBP‐Depolymerase), composed of a material‐binding peptide and polymer‐degrading enzyme, has been reported for PET (Thc‐Cut1‐PBM, 3.75‐fold improved [39]; Dermaseptin S1‐Tfuc2, 22.7‐fold improved [37]; Tfuc2‐LCI, 34.5‐fold improved [40]), Polyester‐PU (Tachystatin, 6.6‐fold improved [14]), and PLA (ICCG‐Cg‐def YH, 1.4‐fold improved [41]). The fusion protein concept (MBP‐Depolymerase) is inspired by naturally occurring enzymes such as cellulases, in which a cellulose‐binding domain (CBD) is often used for sugar‐specific binding and efficient cellulose degradation (amorphous and crystalline) [42]. Interestingly, CBDs have also been reported to bind to PET, with hydrogen‐bond formation important for interfacial recognition [43]. In only one report, an engineered MBP has been reported to accelerate mix‐plastic degradation via engineered MBP‐Fusions proteins (PLA over PS; 1.4‐fold improved) [41].

A class of short peptides (<20 amino acids [35]) that bind to PS and PET originates from phage display experiments. Several small PS‐binding peptides have been reported, characterized by enrichment of aromatic residues at the N‐terminus or a high occurrence of hydrophobic residues [44, 45]. Phage display peptides binding to PET were generally characterized by a low net charge in another study, and the best binding results were achieved with the sequence DEYCCNN [46]. In contrast to PS, aromatic residues are less important for strong PET interactions. In addition to phage display studies, small peptides for PS and PET binding have recently also been designed purely in silico with improved predicted binding characteristics [47, 48]. Molecular simulations that elucidate the interactions between larger MBPs and synthetic polymers have, to our knowledge, only been performed in two reports (PS [49] and PLA [41]). Lessons learned from the first report include increased PS binding resulting from the introduction of aromatic amino acids, which can improve binding strength directly, via polymer interactions, or indirectly, via structural changes. The other study highlighted a change in contact frequency of interfacial residues, mostly caused by structural changes induced by amino acid substitutions.

Based on peptides reported to bind PS [36, 44, 45, 47, 49] and PET [36, 37, 43, 46, 48], we hypothesize that aromatic residues, especially tyrosine, might be the amino acids of choice to achieve material‐specific PET over PS binding.

In this study, a computationally driven approach based on amorphous PET and PS models was used to achieve material‐specific PET over PS binding. Interfacial design of material‐specific binding peptides was attempted by studying aromatic interactions between the MBP MacHis and amorphous PS‐ and PET‐surfaces via MD simulations. MacHis was selected for its high flexibility and ability to adapt to molecular interactions without being restricted by a rigid fold. The high percentage (26%) of histidine residues with a pKa of 6.0 in the peptide structure is a unique feature that promises regeneration of the bound peptide by switching its charge via a pH shift. MacHis has been used to functionalize synthetic polymers for thin film coating, including PET, PA, PP, PE and PVC [50]. The computational investigation identified Tyrosine as the prime amino acid for achieving PET binding over PS binding. Based on computational studies, systematic experiments were conducted in which Tyrosine residues in MBP MacHis were replaced with negatively charged Glutamate; the Tyrosine‐to‐Glutamate substitution was based on simulation results indicating that Glu did not interact with PS or PET. In total, three Tyrosine residues in MacHis were targeted and recombined, and the resulting peptide variants were biophysically characterized by quartz crystal microbalance with dissipation monitoring (QCM‐D) analysis (determination of binding kinetics; recording of adsorption isotherms; confirmation by MacHis‐eGFP fluorescence screening). Finally, the best‐performing variant, MacHis‐Y30E‐Y34E‐Y36E, achieved a 69% reduction in PS binding compared to the MacHis wildtype; steered MD simulations confirmed the role of Tyrosine in PS and PET binding in silico. General applicability was subsequently confirmed by replacing two Tyrosine residues with Glutamate in the material‐binding peptide LCI.

## 2 Results and Discussion

The results and discussion section is divided into three parts. First, interactions of MacHis WT with amorphous PS and PET surfaces were simulated in silico to determine the positions and amino acid interactions that govern PS and PET binding; subsequent wet‐lab characterization using QCM‐D was performed to verify or refute the MD simulations. In the second part, the best‐performing recombination MacHis variant, MacHis_Y30E_Y34E_Y36E, was further characterized by recording adsorption isotherms on both polymers, and results were compared to steered MD simulations to challenge the simulation systems and to validate the amorphous PS and PET surface models. In the third part, the general applicability of the Tyr‐MD workflow was investigated using a structurally distinct material‐binding peptide, LCI, which was also biophysically characterized by QCM‐D.

### 2.1 Interfacial Molecular Interactions of MacHis With Amorphous PS and PET Surfaces

**FIGURE 1:**
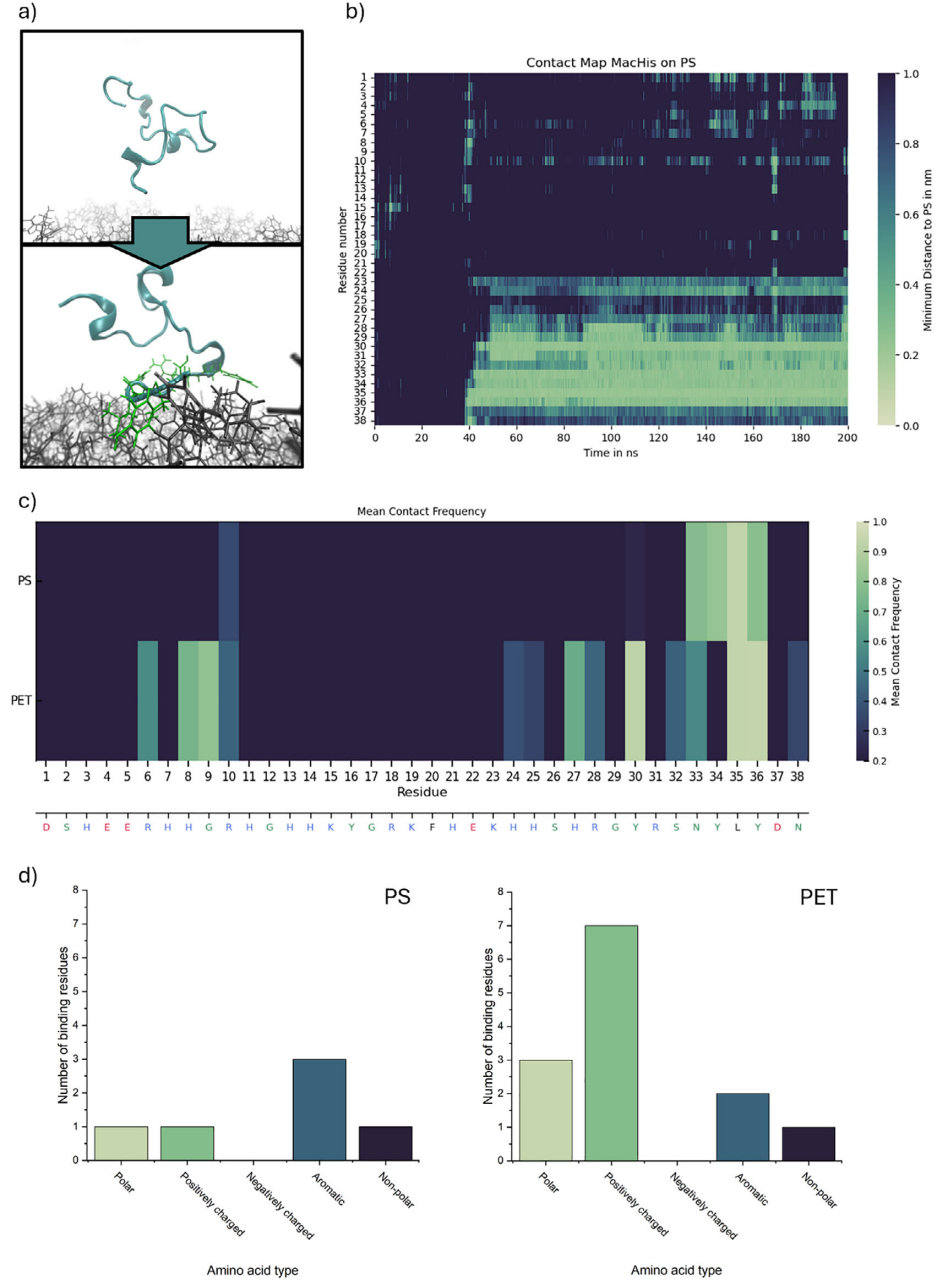
(a) Visual representation of the role of MacHis positions in binding to polystyrene (PS). Interacting Tyrosine residues are shown in green. (b) Contact map for MacHis WT binding to PS over 200 ns of MD simulations. The distance of each residue to the PS polymer over time is indicated by the color shift from dark blue to light green. (c) Contact frequency map for the MacHis WT peptide binding to PS and PET. A contact point is defined by a distance closer than 0.4 nm between any atom of the protein and polymer group. The contact frequency per residue is indicated by the shift from dark blue to light green and represents the mean value calculated from 16 individual simulations for PS and PET. The x‐axis shows the residue number as well as the one letter code for the corresponding amino acid at each position. The color of the letters indicates the chemical type of each amino acid. Red: negatively charged, Blue: positively charged, Green: polar, Black: non‐polar; classification of amino acids type is according to Taylor, 1986 [51]. (d) Aggregated interactions of MacHis WT residues with PS and PET as percentage of all interactions. Interestingly, aromatic interactions are the main interactions in PS and negatively charged amino acid did not show any interactions. In PET aromatic interactions are fewer, in contrast to PS positively charged amino acids are main interactions drivers. Interestingly, also no negative charged amino acid in MacHis (in total five amino acids) showed binding interactions to PET.

Interfacial molecular interactions between MacHis and amorphous models of PS and PET were investigated by performing 16 independent MD simulations for each polymer. Amorphous models of PS and PET were generated using the CHARMM‐GUI polymer builder, which has been used previously to generate PS and PET polymers. Models were generated and equilibrated to achieve realistic polymer densities of 1.09 g/mL for PS and 1.30 g/mL for amorphous PET. Each simulation run was performed for 200 ns under constant temperature (300 K) and pressure (1 bar). The simulation box was composed of the peptide MacHis, a square amorphous polymer surface (10 × 10 nm (PS), 24 × 24 nm (PET)), and ions to control ionic strength, solvated in TIP3P water (up to 450.000 atoms). Starting with random initial rotations of the peptide, its attraction to the polymer surface was observed (Figure 1A). Contact points between MacHis and the two polymers were identified by tracking the MacHis to polymer distance per residue over time (see Methods section), exposing interaction areas of MacHis in a contact map (Figure 1B). The C‐Terminal region of the MacHis showed stable attachment to the PS polymer early in the simulation (after 40 ns) while residues toward the N‐terminus attached after 160 ns. Further analysis was performed by calculating the contact frequency per residue across all 16 simulations on both polymers. A distance between a residue and the polymer below 0.4 nm was defined as a contact. A contact frequency of 1 would indicate a residue that was below the threshold over all simulation datasets, while a frequency of 0 would indicate no contact in any of the MD‐simulations. The region from residue 33 to 36 is pivotal for interactions with PS with additional but weaker contributions at position 10 and 30 (see contact frequency heatmap Figure 1C). MacHis interactions with the amorphous PET model surface yielded 14 residues with contact frequencies between 0.5 and 0.8, which indicate, as a general trend, multiple binding configurations to PET in contrast to the one main interaction area for PS binding (Figure 1C). Involvement of the C‐terminal region for PS binding indicates that Tyrosine 34 and 36 are main contributors to the binding; the latter observation is further fostered by Tyrosine 30, which was identified as further main interaction point using the heat map. MD simulations and literature reports provided the foundation for the proposed hypothesis that Tyrosines 34 and 36 mediate PS contact via π‐π interactions and bring Asparagine 33 and Leucine 35 into close proximity to PS. Interaction patterns of PET binding are more diverse (Figure 1C) and are governed by residues such as Histidine or Arginine (Figure 1C), which are capable of establishing hydrogen bridges. A summary of all interacting residues per polymer is given in Figure 1D. The interaction with PS is primarily governed by six residues, half of which are Tyrosine, with small contributions from a polar, a positively charged, and a non‐polar amino acid. PET binding showed a high frequency of contact for positively charged residues (53,8% of all residues in close contact). 40% of all positively charged amino acids in MacHis were observed to establish contact with the PET surface, while aromatic residues played a lesser role (16.7% of all binding residues). Interestingly, no negatively charged amino acid fell below the 0.4 nm cutoff, which classifies contacts in all 16 simulations on either polymer. This indicates that negatively charged residues are of least importance for binding to PS and PET. These trends imply that PS interactions of MacHis are mainly guided by aromatic interactions, whereas PET interactions rely on positively charged residues, possibly via dipole‐dipole interactions or hydrogen bonds. Negatively charged residues did not seem to facilitate any interactions. Extensive MD simulations yielded the results that Tyrosine residues at positions 30, 34 and 36 in the MacHis peptide present main interaction points with the PS polymer and that the binding is predominantly governed by aromatic interactions. PET binding is mainly shaped by seven positively charged amino acids, which account for 40% of all positively charged residues in MacHis, resulting in a broader patch of the peptide that establishes contact with PET. A comparison to experimental results and a discussion on simulation accuracy can be found in the next section on experimental validation of the role of Tyrosine residues in PS binding.

### 2.2 Experimental Validation of the Role of Tyrosine in PS Binding of MacHis

**FIGURE 2:**
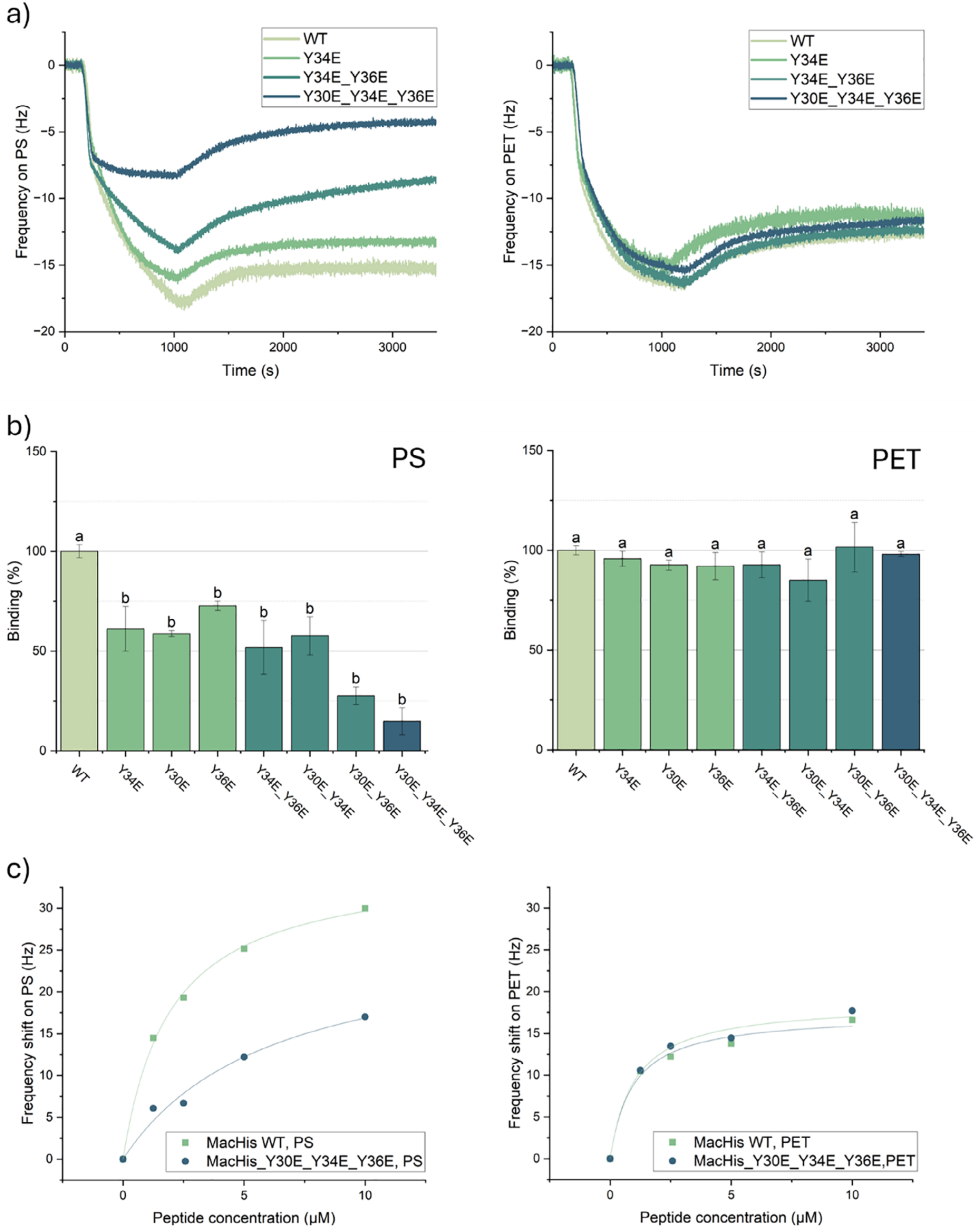
QCM‐D characterization of MacHis variants binding to PS‐ and PET‐coated sensors. a) Frequency shift over time for the direct comparison of variants with increasing number of Y to E substitutions. Peptides were applied for 15 min after a stable baseline was established, and the sample was then washed with binding buffer to remove loosely bound peptides. Measurements were run for 1 h or until a stable plateau was reached during wash. b) Comparison of all single‐, double‐, and triple substitutions possible with positions 30, 34, and 36. The binding was quantified using QCM‐D and the frequency shift was normalized to a wild‐type control that was run in parallel. The bar for each variant shows the average of triplicate measurements. Error bars indicate the standard error. Bars sharing the same letter as WT are not significantly different from the WT. Bars labelled with a different letter from WT are significantly different from the WT (two‐tailed t‐test, *p* <0.05). c) Adsorption isotherms for MacHis WT and the MacHis‐Y30E‐Y34E‐Y36E variant on PS (left) and PET (right) recorded by varying the peptide concentration and measuring the resulting frequency shift on QCM‐D. Data is fitted using a rectangular Hyperbola function that resembles a Langmuir isotherm fit. The parameters F_max_ and b are used to describe the maximum frequency shift and the peptide affinity, respectively.

MD simulation identified the stretch from 30 to 36 as the main interaction region between MacHis and PS, with a key role played by three Tyrosine residues at positions 30, 34, and 36. Site‐directed mutagenesis at these positions in the MacHis‐WT was performed to experimentally confirm that replacing Tyrosine with a non‐interacting amino acid reduces PS binding without, ideally, affecting PET binding. Based on the MD simulation results in Figure 1D, negatively charged amino acids did not appear to be involved in MacHis binding to PS or PET. Therefore, the MacHis variants, MacHis‐Y30E, MacHis‐Y34E, and MacHis‐Y36E, were generated in a tag‐free manner (Figure S1), and their binding compared to the MacHis‐WT was analysed through QCM‐D (Figure 2). The QCM‐D measures the resonance of a polymer‐coated swinging quartz crystal and detects increased mass by a decrease in frequency. The decrease in frequency is proportional to the mass of the bound peptide.

All three single‐substituted variants, MacHis‐Y30E, MacHis‐Y34E, and MacHis‐Y36E, show weaker binding to PS than the WT as determined by QCM‐D measurements. The observed difference in frequency shift confirms the key role of the three Tyrosine residues in PS binding; (see MacHis‐Y34E in Figure 2a; MacHis‐Y30E & MacHis‐Y36E in Figure S2a and b). Substitutions to Aspartic Acid were generated and characterized with the same procedure (Figure S3). The binding to both polymers was affected in a similar way; PS binding reduced upon introducing the negative charge, while PET binding remained unchanged. The difference in the frequency shift for these MacHis variants was less pronounced compared to Glutamate substitutions, so all subsequent experiments were performed with substitution of Tyrosine by Glutamate. Interestingly, all three double substitutions (MacHis‐Y30E‐Y34E, MacHis‐Y30E‐Y36E, MacHis‐Y34E‐Y36E) show further reduced binding to PS compared to the MacHis‐WT and single substitutions (see MacHis‐Y34E‐Y36E in Figure 2a; MacHis‐Y30E‐Y34E & MacHis‐Y30E‐Y36E in Figure S4a and b). The triple substitutions variant (MacHis‐Y30E‐Y34E‐Y36E) has the weakest binding affinity to PS (see Figure 2a) when compared to MacHis‐WT and all other six MacHis variants. Figure 2b (left) summarizes the reduced binding of all variants compared to MacHis‐WT (set to 100%). The relative binding of each variant was determined by dividing the obtained frequency shift after 1 h of QCM‐D measurement by the frequency shift obtained for the MacHis WT within the same round of measurement. This way, all substitutions and their effects on binding performance can be compared in a single overview. The QCM‐D results confirm that the Tyr‐MD workflow is well suited to identify the main amino acids that interact with amorphous PS in MD simulations, and that substituting with the negatively charged Glutamate is an excellent approach to reduce PS‐binding. In addition, the experimental QCM‐D data on PET binding of all seven MacHis variants show no or minor reduction (Only up to 20% reduction for MacHis‐Y34E‐Y36E) of PET binding (Figure 2b right). The experimental QCM‐D data for the PET polymer also align well with computational predictions, which indicate a less important role for aromatic interactions in governing PET binding. The abundance of polar and positively charged residues among the binding residues identified in the simulation, along with the small impact of Tyrosine‐to‐Glutamate substitutions on PET binding, suggests that PET binding is primarily governed by polar interactions, given the dipole nature of the PET polymer. Aromatic interactions with PET are still expected, but the high percentage of polar and positively charged residues in MacHis is thought to compensate for lost aromatic interactions in the generated MacHis variants. In summary, the Tyr‐MD workflow with the amorphous PS and PET models reliably and robustly identifies amino acid positions in MacHis that govern binding to PS. These residues can efficiently be replaced by ‘non‐interacting’ Glutamates without significantly compromising PET binding capacity.

Figure 2c shows the obtained frequency shift at varied concentrations (up to 10 µM) of MacHis‐WT and MacHis‐Y30E‐Y34E‐Y36E on PS (left) and PET (right). Data points were fit using a rectangular hyperbola in the form of a Langmuir adsorption model. The parameters F_max_ and b were determined from the fits for all four peptide‐polymer combinations (see methods section) and are listed in Table 1. The obtained adsorption isotherms show a 69% reduction in binding affinity, which is indicated by the flattening of the isotherm slope (see Tab. 1 F_max_ from 35.4 to 27.3 Hz and b values reduced from 0.51 to 0.16 for PS). The slope of the adsorption isotherms is nearly unchanged for PET (see Tab. 1; Fmax from 18.7 to 17.3 Hz and b values from 0.99 to 1.08). This implies unchanged binding affinity to the PET polymer between the MacHis‐WT and the MacHis‐Y30E‐Y34E‐Y36E variant. Interestingly, for MacHis‐WT and MacHis‐Y30E‐Y34E‐Y36E, saturation is approximately reached at 5 µM, indicating a high affinity for PET. (see. Figure 2c right and Table 1). The obtained values for Fmax and b were used to calculate ΔG of the binding process of each peptide polymer pair which is also included in Table 1.

**TABLE 1:** Resulting parameters from fitting adsorption isotherms to QCM‐D data. F_max_: Maximum frequency shift. b: Affinity parameter describing curve slope.

Peptide‐polymer pair	F_max_ (Hz)	b (µM^−1^)	ΔG (kcal/mol)
WT PS	35.39	0.51	−7.84
Y30E‐Y34E‐Y36E PS	27.27	0.16	−7.15
WT PET	18.68	0.99	−8.28
Y30E‐Y34E‐Y36E PET	17.31	1.08	−8.24

**FIGURE 3:**
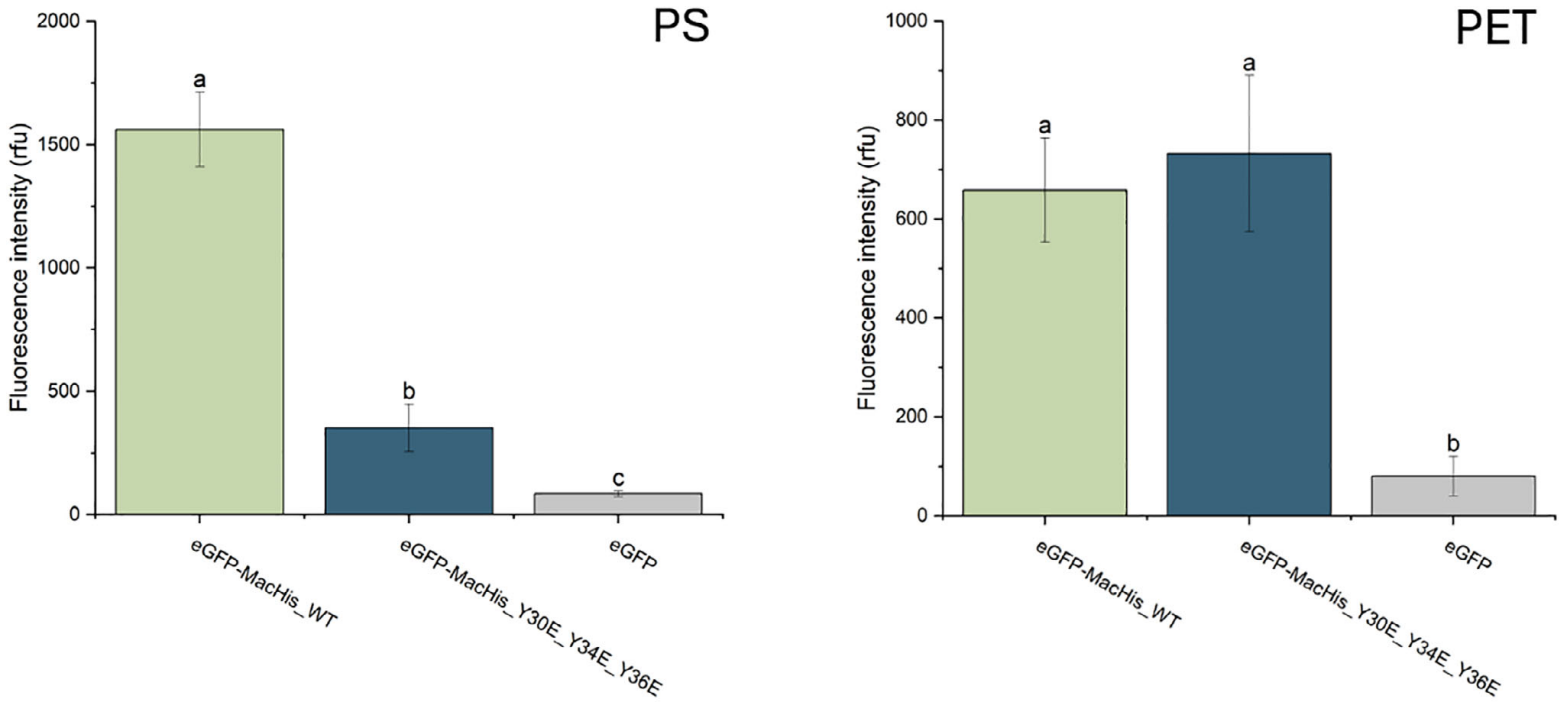
Characterization of MacHis binding to PS (left) and PET (right) using eGFP as a fluorescent reported protein. eGFP and MacHis were produced as a fusion protein and were applied to polymer‐coated MTPs directly from the cell lysate. The cell lysate was diluted to a normalized fluorescence intensity to account for differences in expression. Both polymers were washed five times using Tris/HCl buffer (50 mM, pH 8.0) before the residual fluorescence was recorded. eGPF alone served as a control for binding introduced by the eGFP fusion. Background from buffer and polymer autofluorescence was subtracted in all samples. Bars labelled with the same letter are not significantly different from each other. Bars with different letters are significantly different from each other (two‐tailed t‐test, *p* <0.05).

A further validation of the QCM‐D data for the MacHis‐WT and the MacHis‐Y30E‐Y34E‐Y36E variant was performed by genetic fusion of the genes encoding MacHis WT and the Y30E‐Y34E‐Y36E variant to enhanced green fluorescent protein eGFP with a 17‐amino acid helix spacer, which is reported to efficiently separate the material‐peptide binding domain from the eGFP protein [52]. The eGFP fluorescence was used, as previously reported, to quantitatively compare MBP binding [49, 53]. Purified eGFP alone was used as a negative control to quantify PS and PET binding. Figure 3 summarizes the obtained fluorescence signals for MacHis‐WT and the MacHis‐Y30E‐Y34E‐Y36E on PS (Figure 3 left) and PET (Figure 3 right). In case of PS, a fluorescence intensity drop of 77% (from 1561.66 rfu to 351.33 rfu) is observed, which correlates well with the QCM‐D data (85% drop; Figure 2b). In case of PET, a slightly higher unspecific eGFP adsorption was observed, and the fluorescence intensity values did not change within error‐bars, which also confirms the QCM‐D data (Figure 2b right).

**FIGURE 4:**
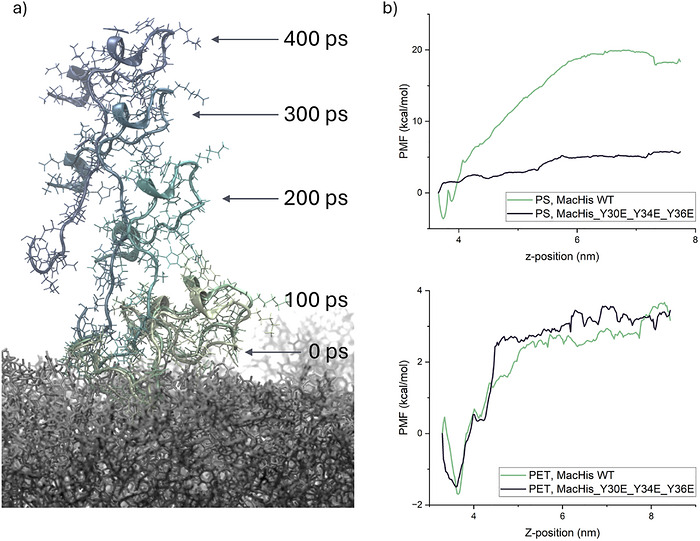
Steered molecular dynamics simulation of MacHis interaction with PS and PET polymers. a) Overview of structures along the pulling trajectory for MacHis WT on PS. Time steps are indicated next to each structure. Frames from this trajectory were isolated at center of mass intervals of 0.2 nm to generate umbrella sampling windows. b) PMF profiles calculated from umbrella sampling for MacHis WT and MacHis‐Y30E‐Y34E‐Y36E on PS (above) and PET (below).

As a final challenge, for the amorphous PS and PET models and the role of Tyrosine in PS binding, steered MD simulations were performed for MacHis‐WT and the MacHis‐Y30E‐Y34E‐Y36E and the PS and PET polymers (see. Figure 4a,b). The radius of gyration obtained from binding simulations with PS indicates that the two peptides adopt distinct bound conformations (Figure S5). This can result from altered peptide conformation due to the triple substitution or from the inherently high flexibility of the coiled peptide, which can adapt to the encountered surface topology. The peptides were pulled from the surface to generate structures for umbrella sampling, with the z‐position of the peptide serving as the reaction coordinate (Figure 4a). The applied forces and the resulting peptide movement over time are shown in Figure S6. A significant difference in the force required to achieve the desired pulling rate was noted on the PS polymer (Figure S6 left). Interestingly, the C‐terminal region of the MacHis‐WT peptide containing residues Y34 and Y36 stayed in contact with the PS polymer the longest (Figure S7). A pulling force of 1000 kJ/mol/nm was observed for the MacHis‐WT on PS at maximum (Figure S6a), which resembles a force scale of breaking 10–30 hydrogen bonds at once, for intuition. Umbrella sampling was performed to quantify the free energy of binding indicated by the potential of mean force along the z position of the peptide in the simulation box (Figure 4B); the obtained results for MacHis‐WT (green line) in comparison to the MacHis‐Y30E‐Y34E‐Y36E (black line) on PS show the binding energy loss caused by the three Tyrosine substitutions to Glutamates. A reduction of binding free energy from 23 to 5 kCal/mol corresponds to a reduction by 69%. The similarity of binding free energy on PET for MacHis‐WT and MacHis‐Y30E‐Y34E‐Y36E underlines the negligible influence of the Tyrosine to Glutamate substitutions on PET interaction. The observed binding free energies from all SMD simulations are consistent with previous studies that simulated binding peptides and their interactions with synthetic polymers [48].

The results from umbrella sampling match the trend observed in adsorption isotherm measurements (Figure 2C). The 69% decrease in binding affinity to PS between MacHis‐WT and MacHis‐Y30E‐Y34E‐Y36E, indicated by the curve slope, can be correlated with the observed reduction in binding strength by 69% calculated in the SMD simulation. The PET model accuracy was confirmed by similar performance of MacHis‐WT and MacHis‐Y30E‐Y34E‐Y36E, which correlates well with the recorded adsorption isotherms on PET (Figure 2C). The combined results from both methods imply that the reduction in Tyrosine content in MacHis drastically reduces the peptide's affinity to PS. This phenomenon highlights the importance of aromatic interactions in addressing the PS polymer. The ΔG shift predicted by MD simulation is much more pronounced compared with the calculated ΔG from QCM‐D experiments. The simulation system is able to predict a change in binding affinity qualitatively, but the computation‐experiment loop is not closed quantitatively.

The obtained results for MacHis‐WT and Tyrosine‐to‐Glutamate substituted variants correlate well with a recently published experimental approach in which the material binding peptide LCI was subjected to site‐saturation mutagenesis at all 47 positions and screened for PS binding [49]. LCI has a very different structure than Mac‐His composed of four anti‐parallel ß‐sheet, whereas MacHis presents a mostly coiled or helical structure. Interestingly, substitutions of aromatic residues were reported to improve PS binding, highlighting the importance of aromatic interactions for PS binding.

### 2.3 Characterization of Tyrosine‐to‐Glutamate Substitutions in Other Peptides

**FIGURE 5:**
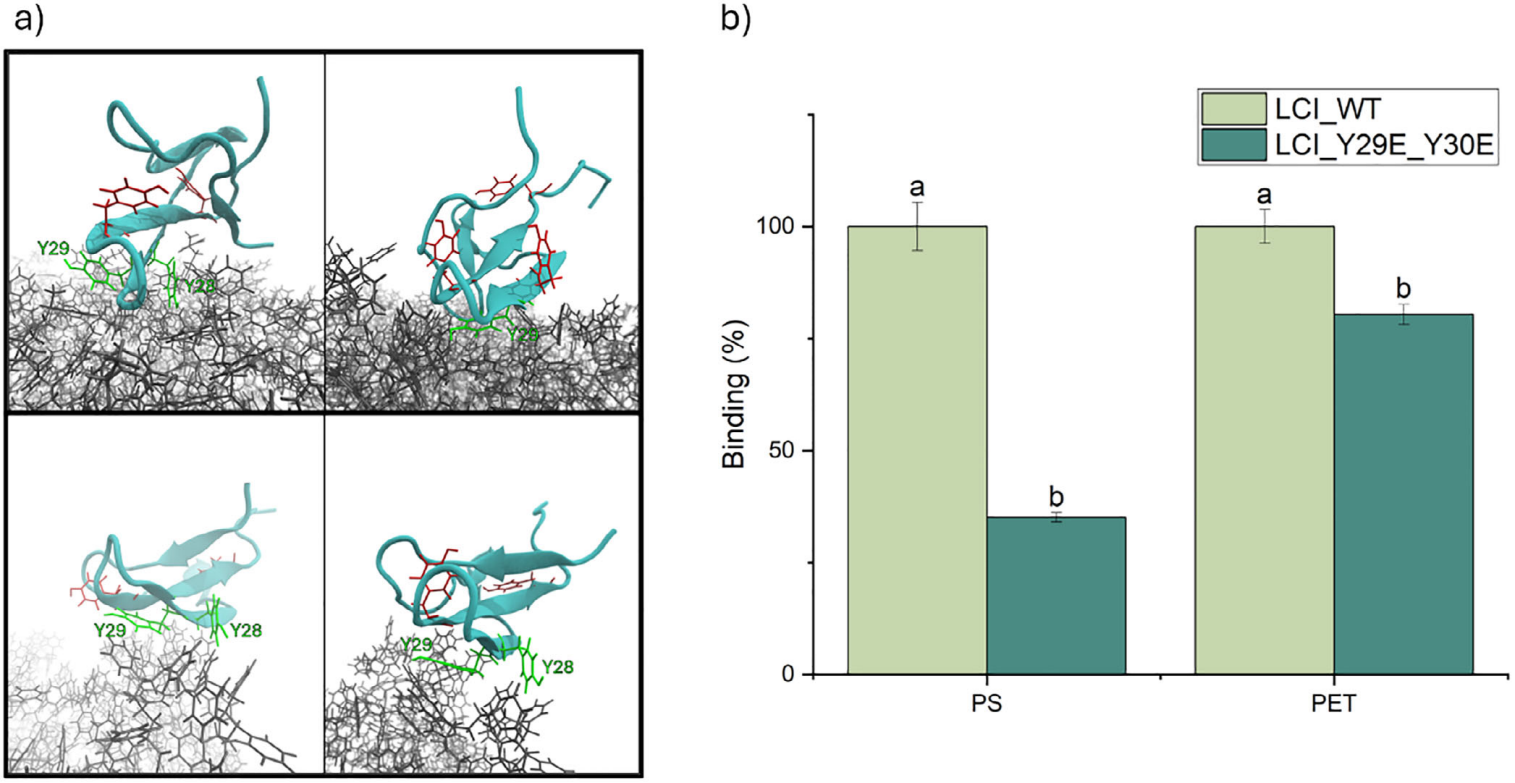
Transfer of Y to E substitutions to the LCI peptide. a) Visualization of Y residues in close contact with the PS polymer. Tyrosines closer than 0.4 nm are shown in green; residues farther away are shown in red. Y29 and Y30 were identified as important Tyrosines. b) Peptide binding to PS and PET measured using QCM‐D. The frequency change indicating binding of the wild‐type peptide was normalized to 100%. The frequency change achieved with the LCI‐Y29E‐Y30E variant is shown relative to the wildtype. All measurements were performed in triplicate. Error bars indicate the standard error of mean. Bars labelled with the same letter are not significantly different from each other. Bars with different letters are significantly different from each other (two‐tailed t‐test, *p* <0.05).

The structurally distinct material‐binding peptide LCI was selected as a use case to assess whether the Tyr‐MD workflow is generally applicable to structurally diverse material‐binding peptides. The binding of LCI‐WT to PS was previously reported to be stronger than to PET [36]. However, a reduction in PS binding without altering PET binding will still elucidate transferability of the engineering approach to another protein fold. In total, 4 MD simulations under the same conditions as those presented for MacHis were performed to identify Tyrosine residues in LCI important for PS binding. Applying the MacHis‐defined selection parameters (contact: d <0.4 nm) identified two out of four Tyrosine residues at positions 29 and 30 as important for PS binding. Tyrosine residues at positions 29 and 30 are located at the end of the longest β‐sheet close to the third loop region of the peptide (Figure 5a). Tyrosine residues 29 and 30 came into proximity of the PS surface in three of four simulations (Figure 5a). The fourth simulation only revealed the contact of Tyrosine 30 with an axial rotation of the β‐sheet, turning Tyrosine 29 away from the PS‐polymer. Based on the MD simulations, both Tyrosine residues were substituted for negatively charged Glutamate (LCI‐Y29E‐Y30E). The LCI WT and LCI‐Y29E‐Y30E were produced and purified as previously described for MacHis, and the binding performances to PS and PET were measured using QCM‐D (Figure 5b).

Figure 5b shows a similar trend for LCI as it was found for MacHis, validating the Tyr‐MD workflow for the structurally distinct LCI peptide. In detail, for PS, a 67% reduced binding affinity of the LCI‐Y29E‐Y30E in comparison to LCI‐WT was obtained. The reduced LCI‐PS binding correlates well with MacHis, which showed a reduction in binding by 50% on average for double Tyrosine substitutions (see Figure 2b left), and an 85% reduction for MacHis Y30E‐Y34E‐Y36E.

**FIGURE 6:**
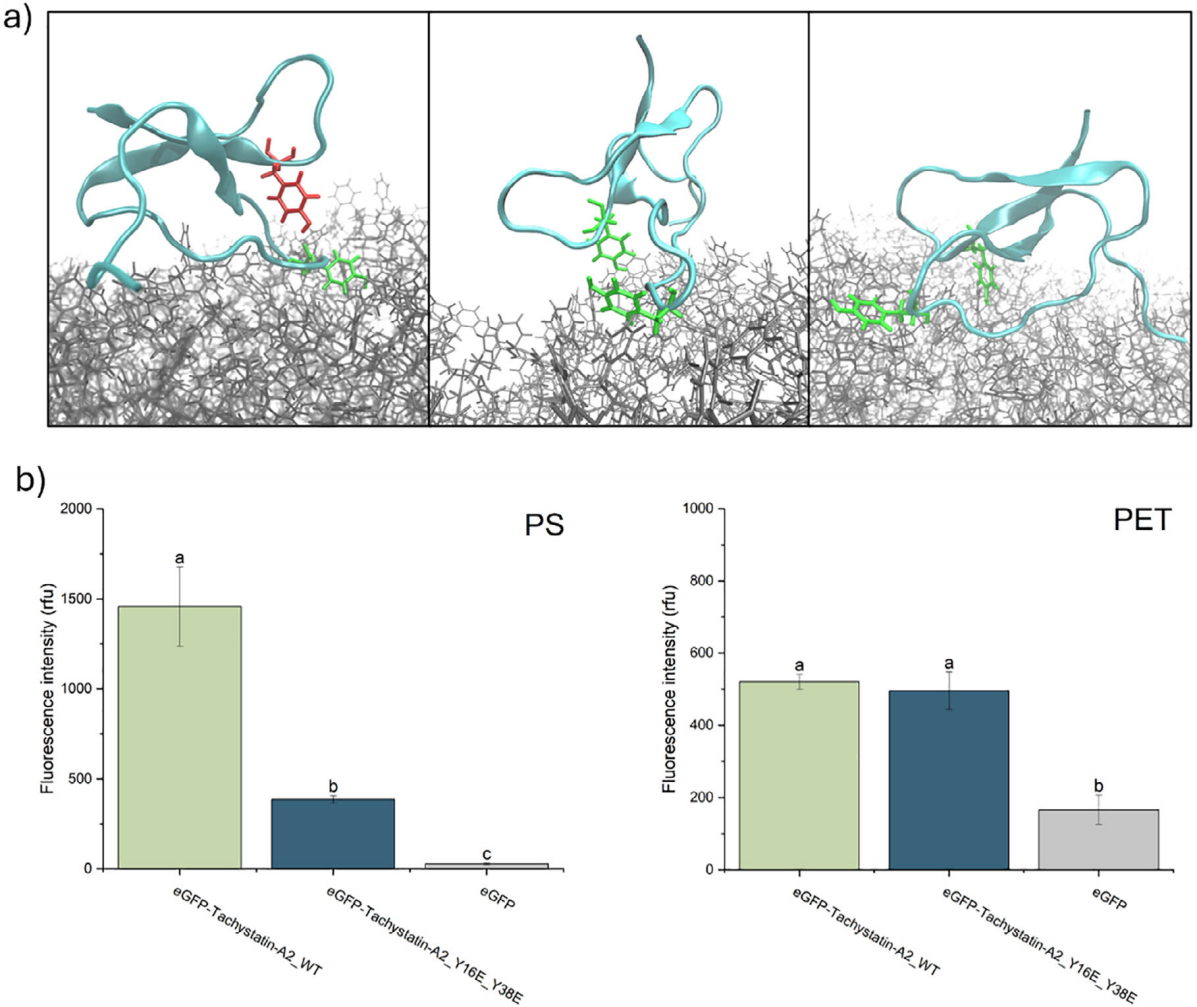
Transfer of Y to E substitutions to the Tachystatin A2. a) Visualization of Y residues in close contact with the PS polymer. Tyrosines closer than 0.4 nm are shown in green; residues farther away are shown in red. Y16 and Y38 were identified as important Tyrosines. b) Peptide binding to PS and PET measured using eGFP fluorescence. All measurements were performed in triplicate. Error bars indicate the standard error of mean. Bars labelled with the same letter are not significantly different from each other. Bars with different letters are significantly different from each other (two‐tailed t‐test, *p* <0.05).

The approach was extended to a third peptide to assess generality. Tachystatin A2 (TA2) was chosen because it combines long loops with β‐sheets, structural elements present in MacHis and LCI individually. Three MD simulations were performed to characterize the molecular interactions between TA2 and the PS polymer surface. The characterization yielded Tyrosines 16 and 38 as important tyrosine residues for the interaction, as they remained in close contact with the PS surface throughout the simulation (Figure 6a). Both structural elements played a role in binding, as Tyrosine 16 is located in a long loop area and Tyrosine 38 is located at the N‐terminal end of the third β‐sheet in TA2. Both positions were subjected to site‐directed mutagenesis to obtain the variant TA2‐Y16E‐Y38E. TA2‐WT and TA2‐Y16E‐Y38E were produced in fusion with eGFP as previously described for eGFP‐MacHis, since tag‐free purification would disrupt intramolecular disulfide bridges. The binding of both variants to PS and PET was quantified through eGFP fluorescence.

Figure 6b shows a similar trend to the fluorescence quantification in Figure 3. The TA2‐Y16E‐Y38E variant shows 74% reduction in PS binding, while PET binding is reduced by 5%. These results are comparable to the 77% reduction in binding that were obtained with the MacHis triple substitution. This correlation underlines the transferability of the Tyr‐MD workflow. By substituting the interfacial Tyrosine residues, the reduced interactions with the PS polymer cause lower peptide attachment, the same effect that was described for the MacHis peptide.

### 2.4 PET Degradation in the Presence of PS

**FIGURE 7:**
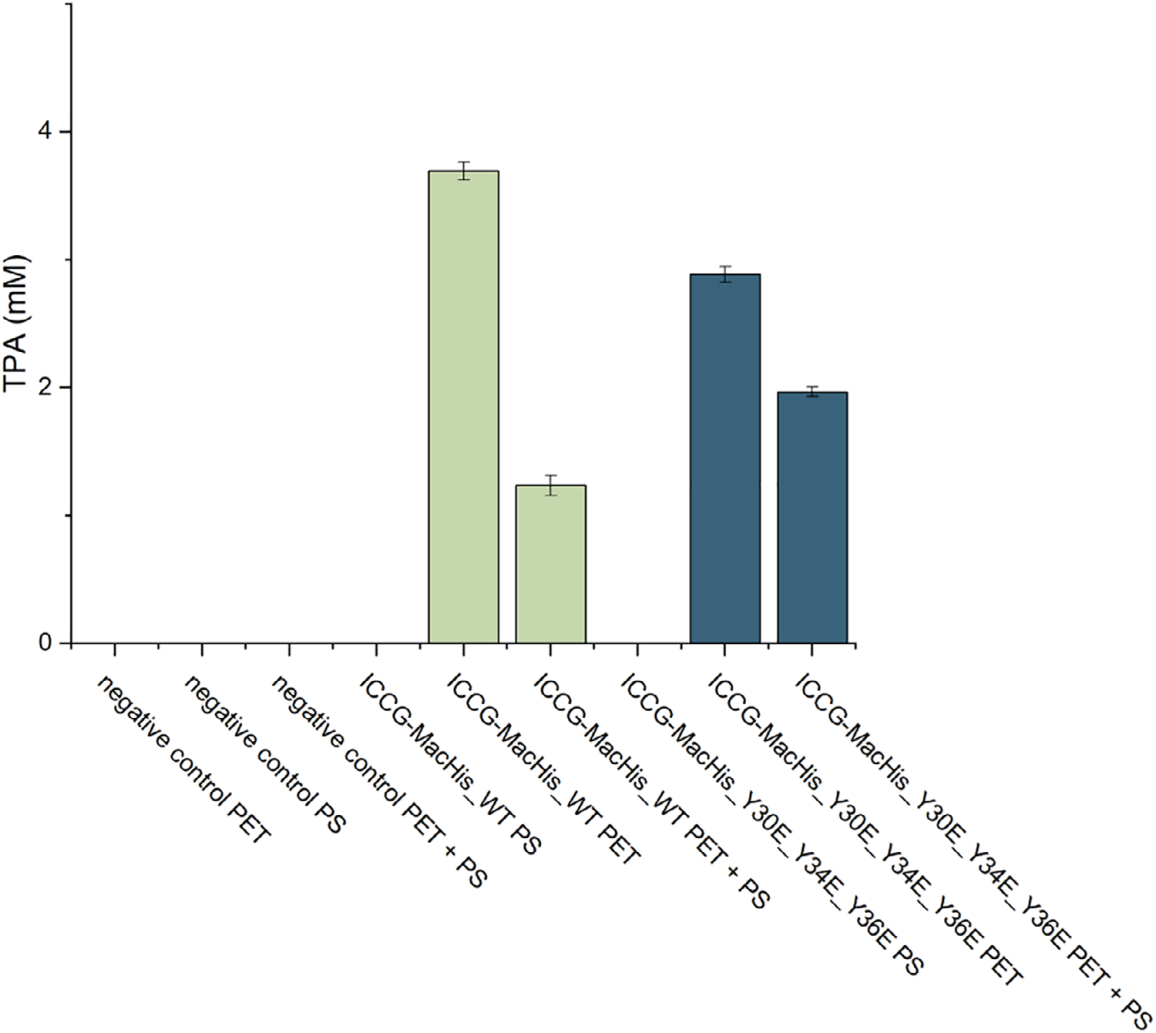
Enzymatic activity analysis of ICCG‐MacHis fusion proteins in pure and mixed plastics. PET film degradation was measured by the terephthalic acid (TPA) produced. TPA formation was detected through TPA hydroxylation. ICCG‐MacHis‐WT and ICCG‐MacHis‐Y30E‐Y34E‐Y36E were compared in their degradation activity in a polymer mixture of equal parts PS and PET as well as in pure polymer samples. Samples without fusion proteins served as negative controls. The degradation reaction was performed at 65°C and 800 rpm for 20 h. One disk of polymer was added to each reaction. Error bars represent standard error of mean of three technical replicates.

The practical application of the engineered material‐binding peptides was tested by PET degradation in the presence of PS. The MacHis‐WT and MacHis‐Y30E‐Y34E‐Y36E peptides were fused to the ICCG variant of the leaf‐branch compost cutinase (LCC) that was engineered for enhanced thermostability and catalytic activity [13]. The fusion proteins were used to degrade 6 mm PET disks punched from PET film. PET degradation was measured by quantifying the produced terephthalic acid (TPA) using a TPA hydroxylation assay [54]. The influence of PS on the reaction was characterized by adding equal amounts of PET and PS to the reaction and comparing the resulting TPA concentration with that of a reaction containing only one polymer (Figure 7). Samples without protein served as a negative control. The activity of ICCG‐MacHis‐WT in the polymer mixture was reduced by 66% compared with the reaction with PET alone. The PET degradation activity of ICCG‐MacHis‐Y30E‐Y34E‐Y36E was in contrast reduced by 31%. These results indicate greater tolerance to the competing PS polymer for the MacHis‐Y30E‐Y34E‐Y36E, as its binding affinity to PS was reduced. The loss of degradation activity in ICCG‐MacHis‐Y30E‐Y34E‐Y36E is likely due to unproductive adsorption onto the PS polymer. The practical applicability of the engineered material‐binding peptide was thereby demonstrated by reduced loss of PET degradation activity in mixed plastic samples compared to pure PET samples.

## 3 Conclusion

The presented MD workflow is suitable for identifying amino acids that govern binding interactions on amorphous PS and PET model surfaces in structurally diverse material‐binding peptides. Initial MD simulations lead to the hypothesis that the aromatic Tyrosine residues are the main driver for PS binding, while PET binding was mediated by polar or positively charged residues. Interestingly, negatively charged amino acids did not form any binding interactions with PS or PET in MD simulations. The hypothesis that the Tyr‐MD workflow generates material‐binding peptides that favor PET binding over PS binding was confirmed in wet‐lab experiments, which showed an 85% reduction in PS binding and a <20% reduction in PET binding. Adsorption isotherms obtained by QCM‐D measurements confirmed the predicted MD‐simulation results, and subsequent umbrella sampling of MacHis‐Y30E‐Y34E‐Y36E, compared with MacHis‐WT, confirmed the reliability and robustness of the simulation systems. Substitution of three Tyrosine residues for Glutamate in the MacHis peptide led to a decrease in binding free energy to PS, which correlated qualitatively with a reduction in binding free energy of 69% observed in SMD simulations. The general applicability of the workflow was tested with the structurally diverse material‐binding peptides LCI and Tachystatin A2, in which the substitution of two MD‐identified Tyrosine residues reduced PS binding by 67% and 74%, respectively. The effect of peptide engineering on PET degradation was shown in an enzymatic depolymerization experiment. Fusing the enzyme LCC_ICCG_ to the MacHis‐Y30E‐Y34E‐Y36E peptide led to a higher residual degradation activity in mixed plastics as compared to MacHis‐WT. In essence, the Tyr‐MD workflow enables the design of binding peptides with improved specificity via MD simulation to target synthetic polymers. The newly generated binding domains for targeting synthetic polymers mimic natural carbohydrate‐binding modules (CBMs), which are used in nature for specific carbohydrate recognition. Binding peptides with improved material specificity that deliver fused enzymes or conjugated chemical catalysts to synthetic polymers are a prerequisite for mixed‐plastic recycling and, more generally, for a circular plastic economy.

## 4 Experimental section

### 4.1 Materials

All chemicals were purchased from AppliChem GmbH (Darmstadt, Germany), Carl Roth GmbH (Karlsruhe, Germany), Fluka (Ulm, Germany), Macherey‐Nagel (Düren, Germany), or Sigma Aldrich Corp. (St. Louis, MO and Deisenhofen, Germany), unless specified. Salt‐free oligonucleotides were obtained from Eurofins Scientific SE (Ebersberg, Germany) and Enzymes from New England Biolabs GmbH (Frankfurt am Main, Germany). Plasmid extraction and polymerase chain reaction (PCR) purification kits were purchased from Macherey‐Nagel GmbH & Co. KG (Düren, Germany). The IMPACT purification kit including the pTXB1 Vector and the chitin resin were purchased from New England Biolabs GmbH (Frankfurt am Main, Germany). The *Escherichia coli* strain BL21‐ Gold (DE3) (Agilent Technologies, Santa Clara, CA) was used for protein expression.

### 4.2 MD Simulation

Molecular dynamics simulations were carried out using GROMACS (version 2021). The CHARMM36 force field was used for peptides and polymers in all simulations. The initial structures of MacHis, LCI and the generated variants were predicted using AlphaFold2 version 2.3.0 with all parameters set to default. To represent experimental conditions, the peptide charge at pH 8 was determined using PROPKA, assigning protonation states. The results were introduced to the peptides representation using the pdb2gmx command in GROMACS. Each peptide structure underwent a two‐part preparation process. First, the structure was simulated in water by solvating it in a triclinic box and minimizing the system's energy using the steepest‐descent algorithm. This was followed by an equilibration in two stages. An NVT ensemble was run for 1 ns with a 1 fs time step and the V‐rescale thermostat at a target temperature of 300 K. An NPT ensemble was run for 100 ps afterward, with pressure maintained at 1 bar using the Parrinello‐Rahman barostat. The production MD run was performed for 100 ns under NVT conditions. In a second step, the resulting structure underwent the same process with the addition of 0.05 m Na and Cl ions to mimic experimental conditions. The final structures from the second production run were extracted and used as input for simulations of both peptides and polymers.

Polymer surfaces were generated using the CHARMM‐Gui polymer builder. This tool provided a method to generate disordered, amorphous polymer melts of PS and PET. The PS structure consisted of 100 oligomeric chains, each with 30 styrene repeats. Atactic polystyrene chains were used as typically found in amorphous PS. The PET structure consisted of 183 chains, each comprising 20 monomeric units.

Peptide‐polymer contacts were studied by placing the peptide 10 Å from the polymer in different orientations. The simulations were performed by running the protocol described above for the peptide. Visualization of the initial and final structures was done using Visual Molecular Dynamics (VMD, Version 1.9.2) [55]. The contact analysis was performed using GROMACS commands *mindist* and *trjconv*.

Pulling simulations were performed by extracting representative bound structure of the peptide from one of the binding simulations. The pull code was added to the simulation parameters with the reaction coordinate being the distance between peptide and polymer along the z‐axis of the simulation box. A pulling rate of 0.01 nm/ps was fixed and the spring constant of the pulling force was set to 1000 kJ/(mol ∙ nm^2^). The pulling force was always applied to the peptide's N‐terminus.

Umbrella sampling was performed by generating a peptide polymer configuration every 0.2 nm along the pulling trajectory. A total of 20 individual simulations were performed along the reaction coordinate for each peptide‐polymer combination. The weighted histogram analysis method (WHAM) [56] implemented to GROMACS was used to calculate the PMF profile along the reaction coordinate.

### 4.3 Variant Generation

Peptide variants were obtained by performing Site‐directed‐mutagenesis. The SDM protocol included the steps described before [57]. Before transformation into *E. coli* BL21‐Gold (DE3), the parental DNA was digested with 20 U of DpnI, and the PCR product was recovered using a PCR extraction kit. Colonies were picked, and successful mutagenesis was confirmed by DNA sequencing.

### 4.4 Protein Production and Purification

Protein expression was performed in 200 mL scale as described before [58]. To increase the production yield, the induction was performed by adding IPTG to a final concentration of 0.4 mM at an OD600 of 0.6 followed by 18‐h incubation at 25°C. Cell were harvested using centrifugation at 3,220 *g* and 4°C for 30 min. The sedimented cells were resuspended in column buffer (20 mM HEPES, 500 mM NaCl, pH 8.5) and after incubation for 30 min at room temperature, cell disruption was achieved by sonication (Vibracell sonicator, 45% Amplitude, 5 min, 10 s on / 10 s off). The lysate was centrifuged at 12,000 *g* and 4°C for 30 min and filtered through a 0.45 µm syringe filter. The clarified lysate was loaded onto a chitin resin column, and the IMPACT‐kit protocol was followed. The column was washed with 10 column volumes of column buffer after the lysate was loaded. Self‐cleavage of the bound protein was induced by washing with 3 column volumes of cleavage buffer (50 mM DTT in column buffer). The flow was stopped overnight, and the target protein was eluted the next day by washing with 3 column volumes of column buffer. Protein samples from the chromatography were checked for purity by sodium dodecyl sulfate‐polyacrylamide gel electrophoresis (SDS‐PAGE). The buffer of pure fractions was exchanged to QCM‐D buffer (50 mM MOPS, pH 7.9) using PD‐10 columns (Cytiva, MA, USA). Protein samples were flash‐frozen in liquid nitrogen and then stored at ‐20°C in frozen aliquots. Protein samples were freshly thawed for analysis and protein concentration was measured using Bradford assay (Pierce Coomassie Plus, ThermoFisher Scientific, Wesel, Germany).

### 4.5 Quartz Crystal Microbalance With Dissipative Monitoring

QCM‐D gold sensors were coated with polymers to measure peptide polymer binding. Residual organic matter was stripped from the chips by 30 min of incubation in a mixture of three parts concentrated sulfuric acid and one part 30% WT hydrogen peroxide. The sensors were rinsed with water and dried with a stream of nitrogen. The sensors were coated by spin coating, for which PS was dissolved at 0.2% (w/v) in chloroform, and PET was dissolved at 1% (w/v) in a mixture of equal parts of chloroform and TFA. The spin‐coating process was performed at 3000 rpm for 1 min, with 150 µL of dissolved polymer added to each sensor. Sensors were dried for 1 h at 23°C or 30°C for PS and PET respectively. Measurements in air before and after coating served as a quality control to ensure consistent film thickness for peptide binding analysis. The peptide concentration was adjusted to 5 µM for every binding experiment. Every QCM‐D experiment was started by flowing binding buffer through the measurement chamber at 1 mL/min until a stable baseline was reached. Measurements were started by applying the peptide solution which reached the measurements chambre after about 5 min at this flowrate. For comparison of peptide variants, the peptide was applied for 15 min followed by washing with binding buffer to remove unbound or weakly bound peptides. The wash phase continued for at least 1 h, or until the frequency shift reached a stable plateau. Since all experiments were run on a four‐channel QCM‐D, each variant was tested alongside a wild‐type sample as a reference to minimize the influence of experimental conditions. Adsorption isotherms were recorded by applying 4 different concentrations of the same peptide to the four sensors during the same run. This time, the peptide application was not limited to 15 min; it continued until the frequency shift reached a plateau. Data from QCM‐D measurements was analysed with the shipped QSense Dfind application.

### 4.6 Adsorption Isotherms

To determine peptide affinity, the QCM‐D adhesion data were plotted against peptide concentration in OriginPro 2024. The curves were fitted with a rectangular hyperbola, similar to the fit used in Langmuir adsorption isotherms (Equation 1). The parameters F and b can be used to describe the maximum frequency shift and the curve slope, respectively, where f is the observed frequency shift and c is the peptide concentration. The frequency shift was seen proportionally to the amount of bound protein, and a steep slope indicated high peptide affinity.

(1) $$f=\frac{F_{max}\cdot b\cdot c}{\left(1+b\cdot c\right)}$$

### 4.7 eGFP Binding Assay

Binding peptides fused to enhanced green fluorescent protein (eGFP) to measure binding through residual fluorescence. The protein fusion included a 17x helix to provide spatial separation of binding peptide and fluorescent reporter protein. Fluorescent screening was performed in MTPs coated with the polymer. The MTPs were coated by adding 200 µL of polymer solution to the well and evaporating the solvent at 50°C. The coated plate was dried overnight and rinsed with binding buffer (50 mM Tris/HCl, pH 8.0). The eGFP fusion proteins were produced as described above and binding experiments were performed with crude cell extract. For this, a section of the cell pellet was taken and resuspended in 40 µL per 100 mg of cells. Sonication was performed as described above and cell debris was removed by centrifugation. The fluorescence of the bright green supernatant was measured at 520 nm using the CLARIOstar plate reader (BMG LABTECH, Germany). The supernatant samples were diluted in binding buffer to achieve a unified fluorescence signal of 15,000 rfu. 100 µL of the dilution was applied to the coated MTP for each sample in triplicate. The MTP was incubated at room temperature and 600 rpm for 10 min. The wells were washed four times with 200 µL of binding buffer with an incubation of 5 min at 600 rpm and room temperature after each buffer application. The liquid was removed from the well and residual fluorescence was measured at 520 nm. Genetically unfused eGPF was used as a negative control.

### 4.8 PET Degradation Assay

The degradation of amorphous PET film was measured by quantifying the TPA produced. The PET and PS films were punched into 6 mm disks with a weight of 5 mg using a pneumatically aided manual press. The disks were washed with ethanol and water and incubated for 10 min in a sonication bath before drying them at 37°C. The degradation reactions were performed by combining one disk per polymer with 500 nM of protein in 500 µL of 0.1 m sodium phosphate buffer with pH 8. The reaction mixture was incubated at 65°C and 800 rpm for 20 h. The produced TPA was quantified by fluorescence measurements of hydroxylated TPA (2‐hydroxyterephthalate (2‐HOTP)). Hydroxylation of TPA was achieved through a Fenton‐like reaction as reported elsewhere [54]. 2‐HOTP fluorescence can be excited at 318 nm, and emission can be recorded at 420 nm. The final TPA concentration was determined from the fluorescence intensity using a TPA calibration curve from 0,01 to 1 mM.

## Author Contributions

**Julian Luka**: conceptualization, Writing – original draft, investigation, methodology, validation, visualization, Writing – review and editing, software, project administration, data curation. **Jonas Berger**: methodology, validation, software, writing – review and editing, data curation, investigation. **Marian Bienstein**: supervision, project administration, writing – review and editing, funding acquisition, conceptualization. **Ulrich Schwaneberg**: conceptualization, writing – original draft, funding acquisition, project administration, supervision, resources, writing – review and editing.

## Conflicts of Interest

The authors declare no conflicts of interests.

## Supporting information




**Supporting File**: advs77104‐sup‐0001‐SuppMat.docx.

## Data Availability

The data that support the findings of this study are available from the corresponding author upon reasonable request.
