# Supplementary material for: Toward Material‐Specific Peptides From in Silico Guided Peptide Engineering to Target PET in the Presence of PS
Source: Adv Sci (Weinh). 2026 Aug 18:e77104. Online ahead of print. doi: 10.1002/advs.77104 (PMC13482935; doi:10.1002/advs.77104)
Supplement: Supplementary file 1 [file ADVS-9999-e77104-s001.docx]

# Toward material-specific peptides from *in silico* guided peptide engineering to target PET in the presence of PS

*Julian Luka , Marian Bienstein, Jonas Berger, Ulrich Schwaneberg**

Julian Luka, Marian Bienstein, Jonas Berger, Ulrich Schwaneberg

Lehrstuhl für Biotechnologie, RWTH Aachen University, Worringerweg 3, 52074 Aachen, Germany

Email: u.schwaneberg@biotec.rwth-aachen.de

Ulrich Schwaneberg

DWI-Leibniz Institut für Interaktive Materialien, Forckenbeckstrasse 50, 52074 Aachen, Germany

Keywords: protein engineering, polymer recycling, molecular dynamics simulations, polymer interfaces, material binding peptides

# Supplementary Information

Supplementary Figure 1: SDS-PAGE of IMPACT purification fractions. M: Spectra Low Range Protein Marker; 1: cell lysate; 2: column flowthrough; 3: column wash; 4-6: elutions fractions 1-3

Supplementary Figure 2: comparative binding experiments assessing MacHis WT, MacHis Y30E and MacHis Y36E. a) Binding of MacHis-WT and MacHis-Y30E to PS (left) and PET (right).b) Binding of MacHis-WT and MacHis-Y36E to PS (left) and PET (right). Binding experiments were always performed with one WT sample as a reference to compare all variants in the end, even though not all could be measured in parallel.

Supplementary Figure 3: comparative binding experiments assessing MacHis-WT, MacHis-Y30E, MacHis-Y34E and MacHis-Y36E. Binding to PS (left) and PET (right) was measured using QCM-D. Binding experiments were always performed with one WT sample as a reference to compare all variants in the end, even though not all could be measured in parallel.

Supplementary Figure 4: comparative binding experiments assessing MacHis WT, MacHis Y30E_Y34E and MacHis Y30E_Y36E. a) Binding of MacHis-WT and MacHis Y30E_Y34E to PS (left) and PET (right). b) Binding of MacHis-WT and MacHis Y30E_Y36E to PS (left) and PET (right). Binding experiments were always performed with one WT sample as a reference to compare all variants in the end, even though not all could be measured in parallel.

Supplementary Figure 5: Radius of gyration of MacHis-WT and MacHis-Y30E-Y34-Y36E during the binding simulation with PS.

Supplementary Figure 6: Progression of applied force and z position of the peptide throughout the pulling simulation. a) MacHis WT on PS; b) MacHis-Y30E-Y34E-Y36E on PS; c) MacHis WT on PET; d) MacHis-Y30E-Y34E-Y36E on PET

Supplementary Figure 7: Contact map of MacHis WT pulled from the PS surface. The distance of each residue from the polymer is shown over 410 ps.
